# Supplementary figures and images for: Neighbors and relatives: How do speech embeddings reflect linguistic connections across the world?
Source: PLoS One. 2025 Aug 25;20(8):e0330755. doi: 10.1371/journal.pone.0330755 (PMC12377560; doi:10.1371/journal.pone.0330755)

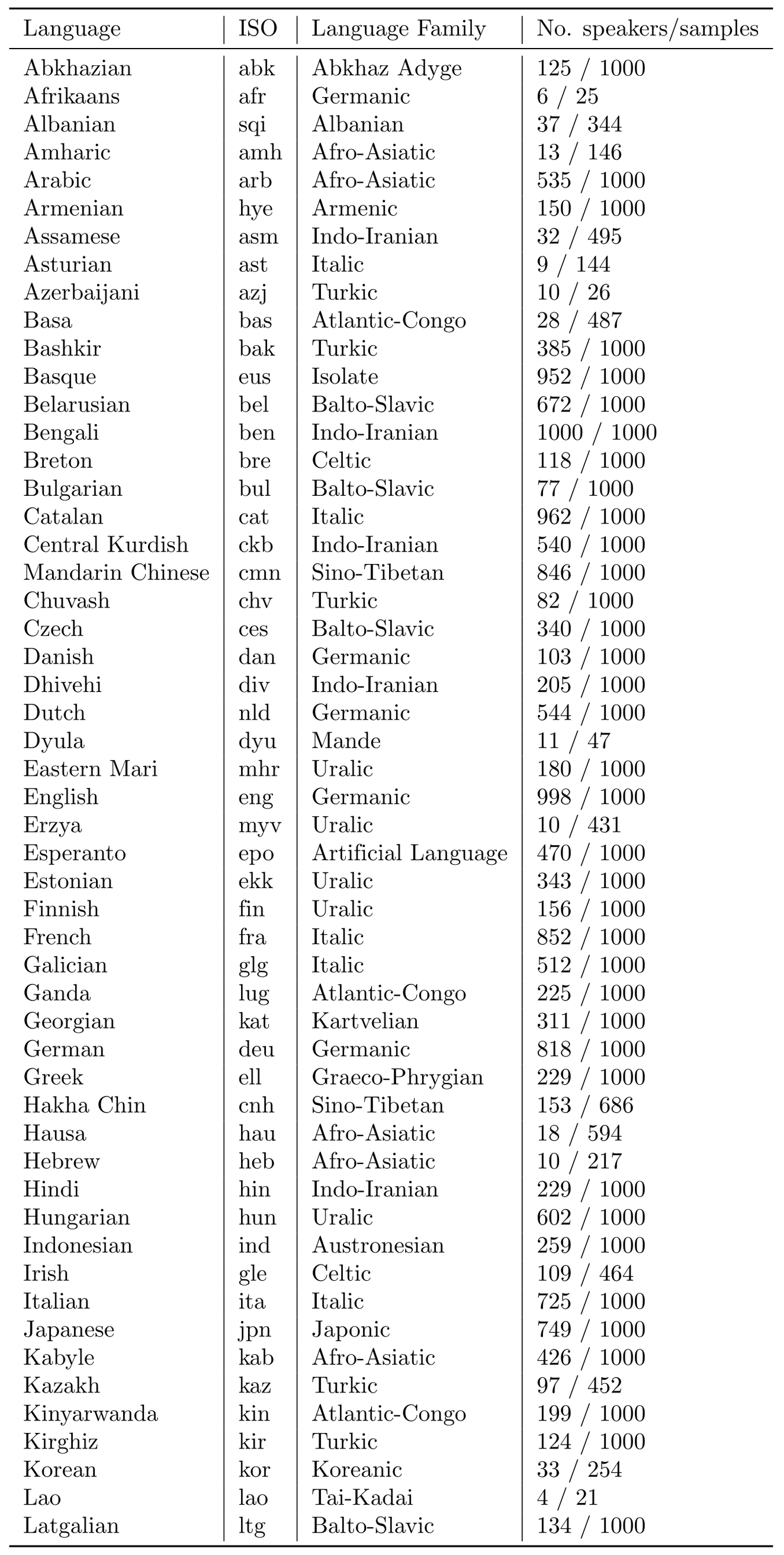

Supplement: S1 Fig — (TIF) [file pone.0330755.s001.tif]

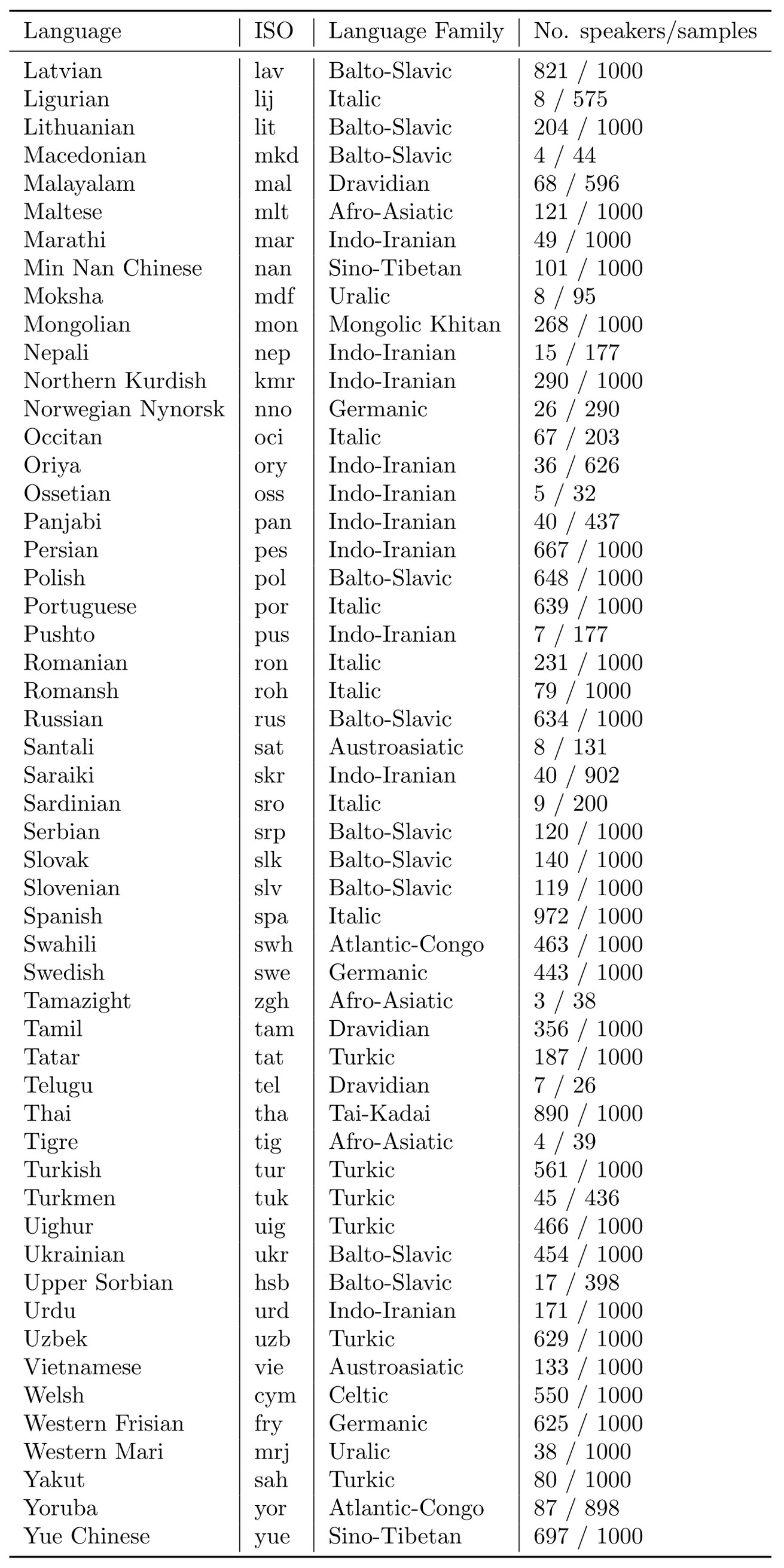

Supplement: S2 Fig — (TIF) [file pone.0330755.s002.tif]

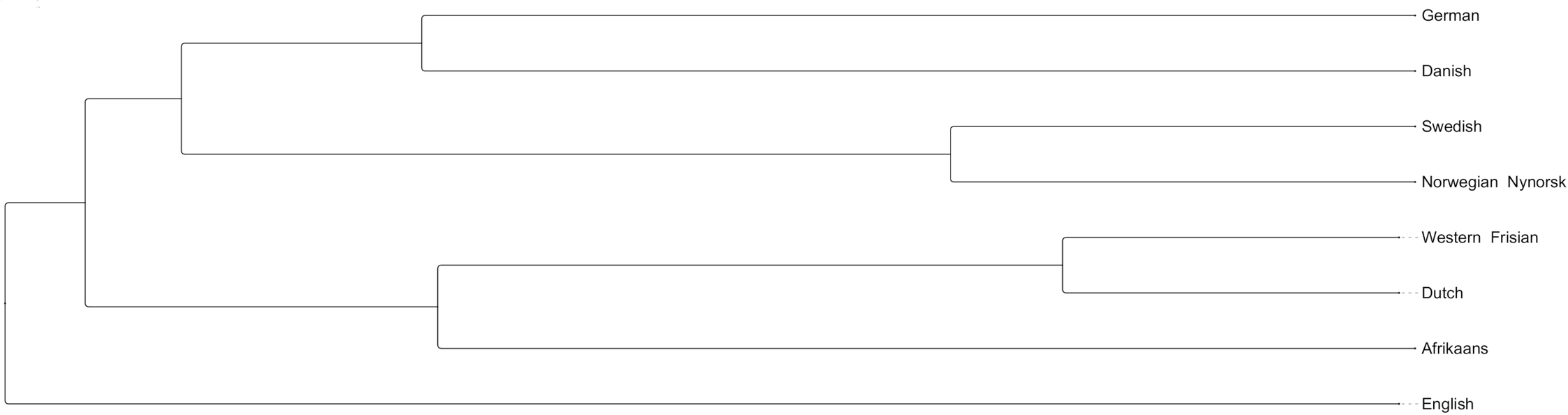

Supplement: S3 Fig — (TIF) [file pone.0330755.s003.tif]

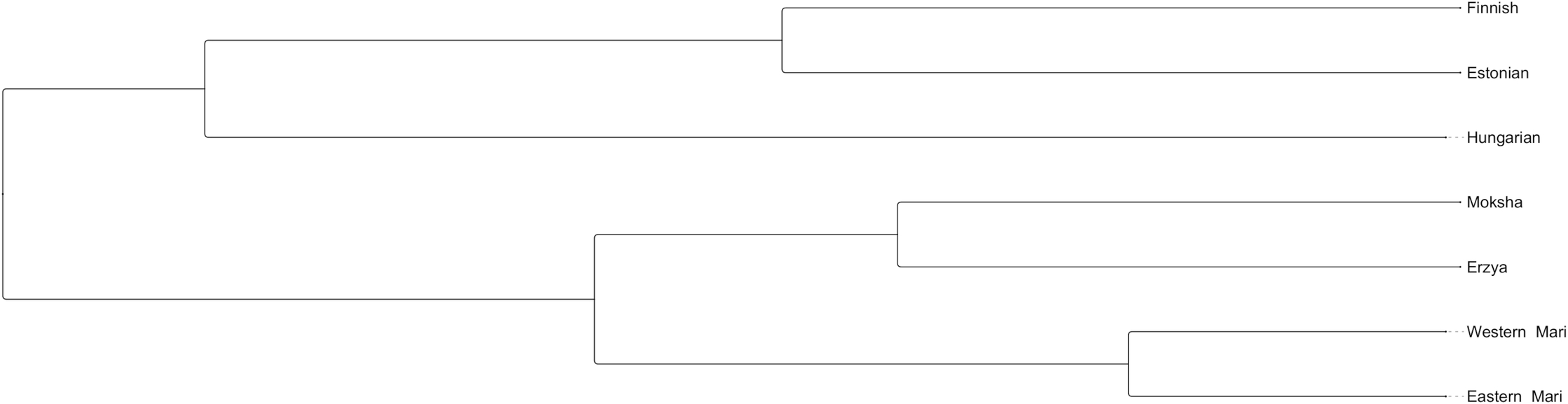

Supplement: S4 Fig — (TIF) [file pone.0330755.s004.tif]

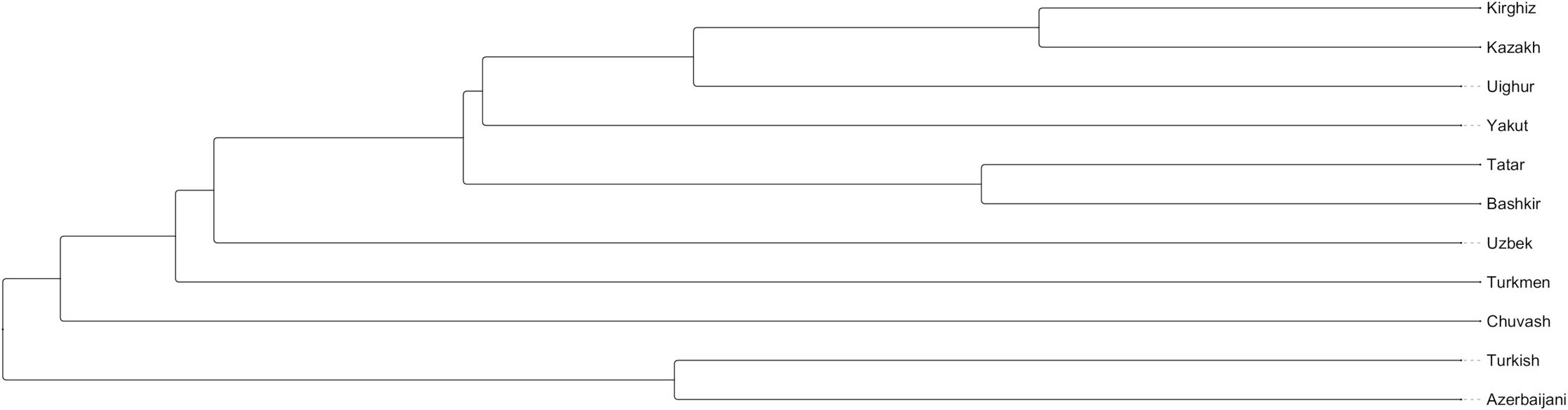

Supplement: S5 Fig — (TIF) [file pone.0330755.s005.tif]

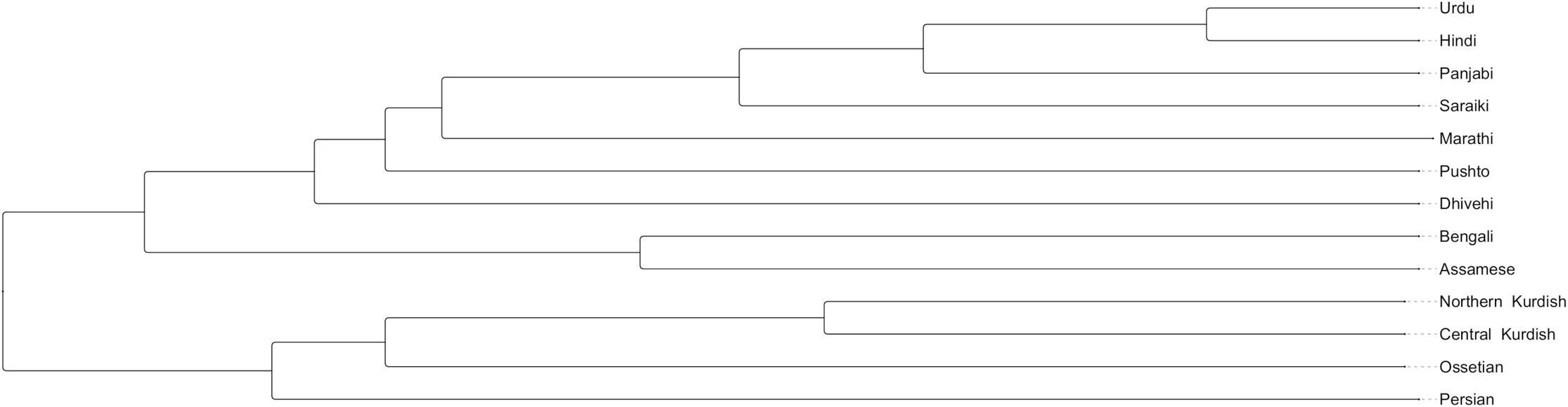

Supplement: S6 Fig — (TIF) [file pone.0330755.s006.tif]

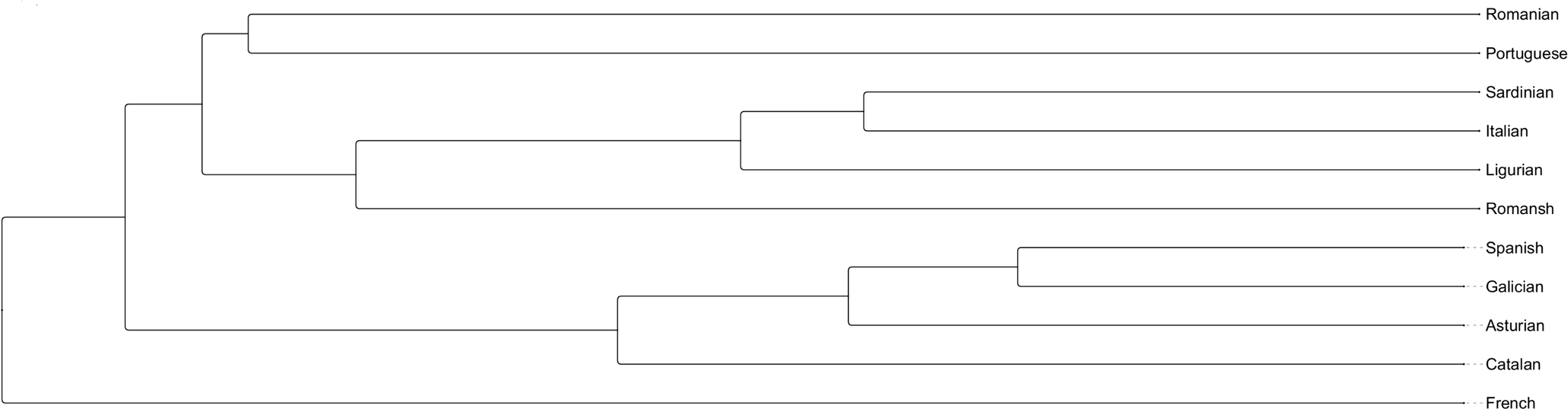

Supplement: S7 Fig — (TIF) [file pone.0330755.s007.tif]

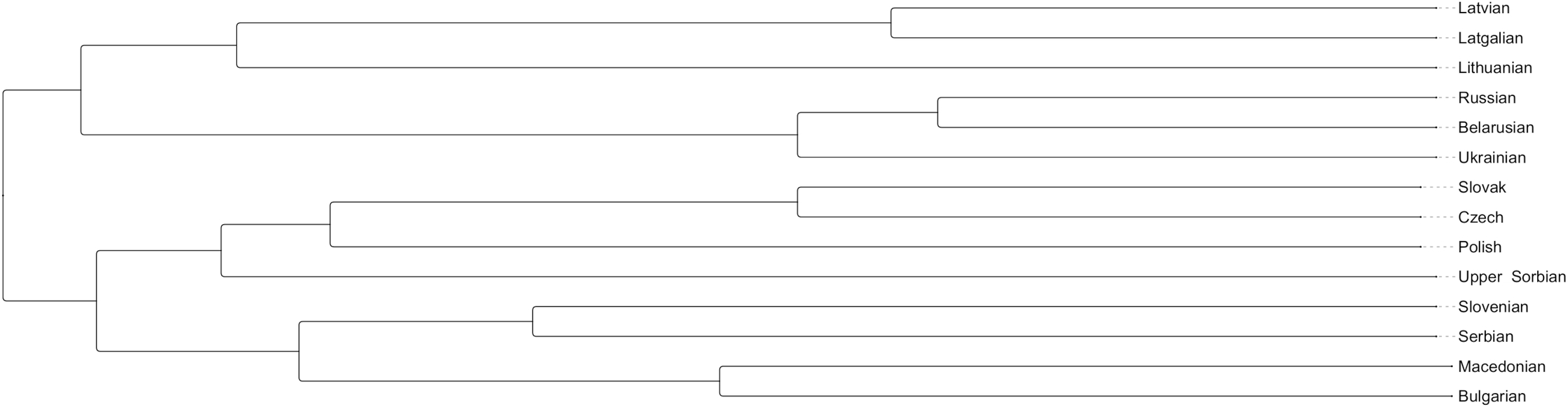

Supplement: S8 Fig — (TIF) [file pone.0330755.s008.tif]

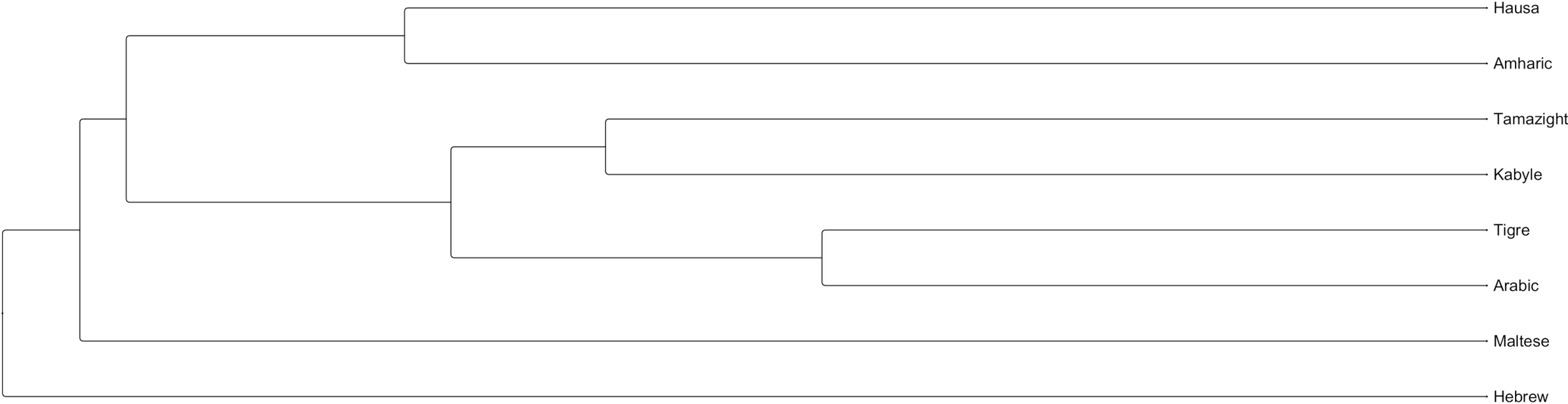

Supplement: S9 Fig — (TIF) [file pone.0330755.s009.tif]
